# Supplementary material for: Alternating Lenvatinib and Trans-Arterial Therapy Prolongs Overall Survival in Patients with Inter-Mediate Stage HepatoCellular Carcinoma: A Propensity Score Matching Study
Source: Cancers (Basel). 2021 Jan 5;13(1):160. doi: 10.3390/cancers13010160 (PMC7796435; doi:10.3390/cancers13010160)
Supplement: Supplementary file 1 [file cancers-13-00160-s001.pdf]

cancers

MDPI

# Supplementary materials: Alternating Lenvatinib and Trans-Arterial Therapy Prolongs Overall Survival in Patients with Inter-Mediate Stage HepatoCellular Carcinoma: A Propensity Score Matching Study

**Shigeo Shimose, Hideki Iwamoto, Masatoshi Tanaka, Takashi Niizeki, Tomotake Shirono, Yu Noda, Naoki Kamachi, Shusuke Okamura, Masahito Nakano, Hideya Suga, Taizo Yamaguchi, Takumi Kawaguchi, Ryoko Kuromatsu, Kazunori Noguchi, Hironori Koga and Takuji Torimura**

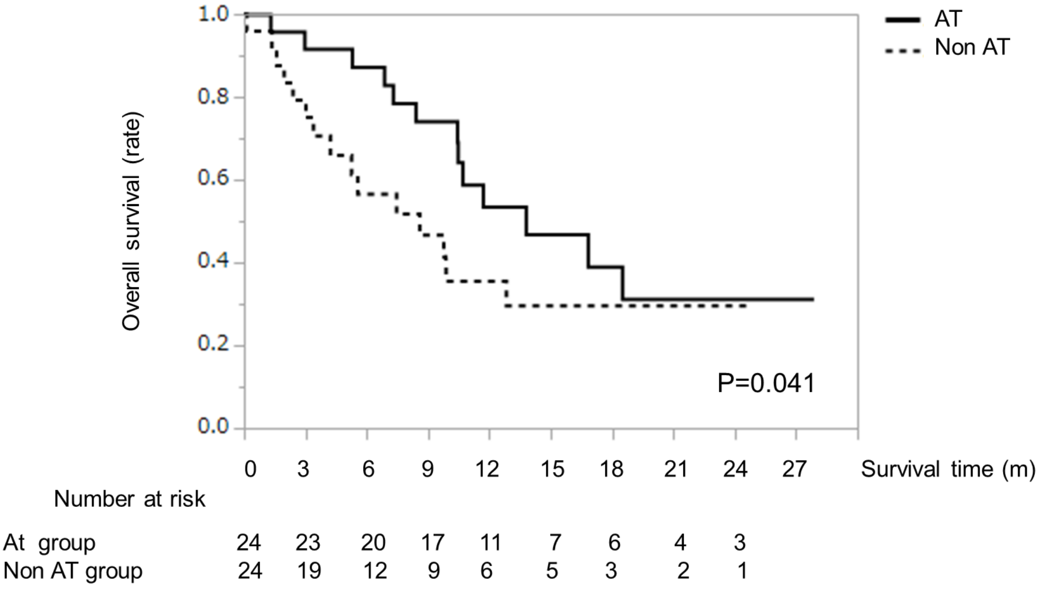


**Figure S1.** Administration period of LEN in AT and non-AT groups after PSM. Kaplan-Meier curves for administration period according to the AT group and the no-AT group. The black line indicates the AT group. The black dotted line indicates the no-AT group. Abbreviation: LEN, lenvatinib; AT, alternating LEN and trans-arterial therapy; PSM, propensity score matching.

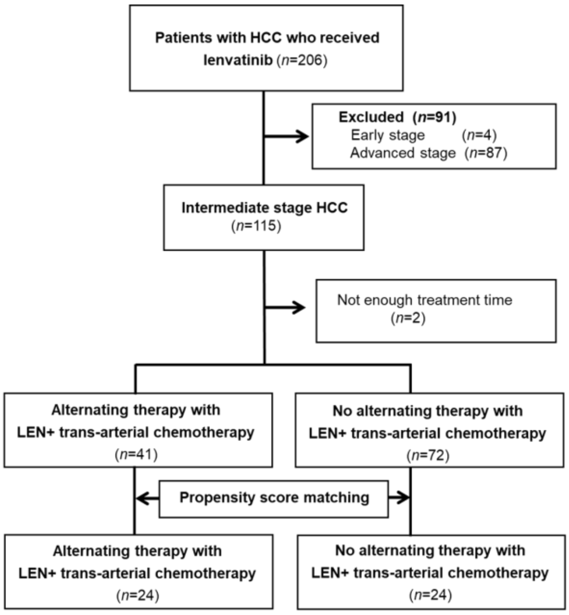


**Figure S2.** Study design. A total of 206 patients with HCC were enrolled from March, 24, 2018 and August, 31, 2020. In the course of the study, 93 patients were excluded, and 113 patients with HCC were analyzed in this study. Then, the data from the 113 HCC patients were applied to propensity match scoring and 48 HCC patients were used for the evaluation. Abbreviation: HCC, hepatocellular carcinoma.

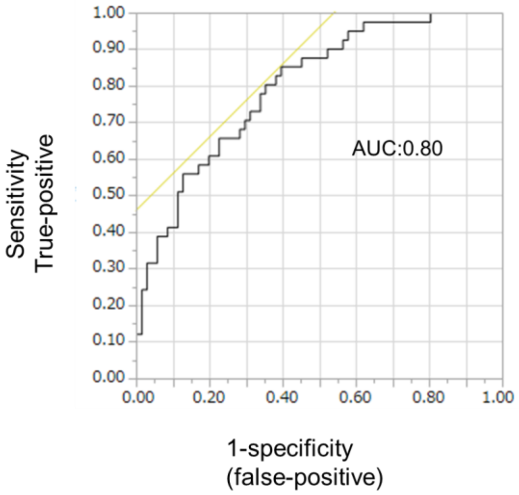


**Figure S3.** C-statistics using ROC analysis. The c-statistics is 0.80, indicating the good ability of the propensity score model to predict the effect of treatment on patients' prognosis.

**Table S1.** Adverse events associated with each treatment (Grade ≥ 3).

| Adverse event | AT ($n$ = 41) | Non-AT ($n$ = 72) | $P$-value |
|---|---|---|---|
| Hypertension | 17.0% (7/41) | 16.7% (12/72) | 0.955 |
| Urine protein | 12.2% (5/41) | 15.2% (11/72) | 0.651 |
| HFSR | 7.3% (3/41) | 2.8% (2/72) | 0.259 |
| Fatigue | 4.9% (2/41) | 9.7% (7/72) | 0.363 |
| Diarrhea | 4.9% (2/41) | 11.1% (8/72) | 0.262 |
| Loss of appetite | 2.4% (1/41) | 1.4% (1/72) | 0.684 |
| Hemorrhage | 0.0% (0/41) | 0.0% (0/72) | n.s |

Abbreviations: HFSR, hand-foot-skin-reaction.
